# Odisha Revisited: A Personal Account

**DOI:** 10.3389/fmed.2021.745337

**Published:** 2021-10-28

**Authors:** Graham R. Serjeant, Andreas E. Kulozik, Beryl E. Serjeant

**Affiliations:** ^1^Sickle Cell Trust (Jamaica), Kingston, Jamaica; ^2^Department of Pediatric Oncology, Hematology and Immunology, University of Heidelberg, Heidelberg, Germany

**Keywords:** sickle cell, India, Jamaica, cohort study, haplotype comparison

## Abstract

In 1986, a paper in the Lancet was the first to collate hematology, molecular findings, and clinical features of homozygous sickle cell (SS) disease in India. The paper came from the group organized by Professor Bimal Kar in Burla Medical College, Sambalpur University, in western Odisha. Although widely quoted, few readers will be aware of the history of this work that is now attached in an informal summary.

## Background

Following the first report of the sickle cell gene in India (1), many surveys established the distribution of the sickle hemoglobin gene recently summarized by Hockham et al. (2). Latterly, much has been learnt on its molecular characteristics from Dr. Roshan Colah and colleagues at the Indian Council for Medical Research (ICMR) Immuno-hematology Unit in Mumbai. In the mid-1980's, Professor Kar and his pediatric colleague, Professor Satapathy had noted the frequency of children with jaundice, joint pains, and a positive sickle test, but although SS disease was suspected, confirmatory hemoglobin electrophoresis was not available at that time at Burla Medical College. Professor Kar sought help from the British Council in Kolkata who responded by sending a statistician, Richard Hayes from the London School of Hygiene and Tropical Medicine. It was a happy choice since although Professor Kar and his colleagues, did not at that time have data for analysis, Richard had worked with the Sickle Cell Unit of the Medical Research Council at the University of the West Indies in Kingston, Jamaica, and recommended that staff from there should visit Burla. Professor Graham Serjeant (Director of the MRC Laboratories in Jamaica) and Beryl Serjeant (chief technologist) were therefore invited by the British Council to visit India in February 1985. The Council arranged a lecture tour that included visits to the ICMR Unit in Mumbai, The University of Pune, the Medical College at Kolkata, and the School of Tropical Medicine where Professor Serjeant gave the 13^th^ JB Chatterji, Memorial Oration entitled “The Broad Spectrum of Sickle Cell Disease.” Finally, after an overnight train journey from Kolkata, they reached Burla Medical College where they saw many children, subsequently confirmed to have SS disease in samples carried back to Jamaica.

To learn more about the patients of Professor Kar, it was proposed to carry a laboratory of equipment and consumables generously loaned/donated for the study by companies in the United Kingdom. The British Medical Research Council's base in Mill Hill, London, oversaw the shipping of six large crates weighing 334 kilograms, to Kolkata where they were transferred to a British Council's Land Rover and traveled with Graham Serjeant and the Nepali driver of the British Council for the 2-day journey to Burla. Meanwhile the visiting team of colleagues that included Dr. Andreas Kulozik, a German research fellow working with the Department of Hematology in Oxford, England, his wife Martina, a dermatologist, who was invaluable in taking blood samples, Su Sirr, an English nurse from the University Hospital in Kingston, Jamaica, who volunteered to work with us, and Beryl, traveled by overnight train to the station at Sambalpur Road, where they were collected by our hosts. There were no hotels in Burla, and we stayed in the Hirakud Dam Rest House First Class built to accommodate the German engineers involved in the construction of the 27 km Hirakud Dam across the Mahanadi River, the largest earth dam in the world. Entertainment was limited and the focus tended to be the European wine shop and the café in the market that had recently installed an Italian espresso coffee machine.

In the medical college, the laboratory was set up in a lecture theater where we had electricity most of the time and cleaning of the glassware was performed by Beryl in the buckets of water of increasing cleanliness until the last bucket contained distilled water. The work in earnest began on Monday, February 24, 1986, when the local radio station announced that a team of international specialists in sickle cell disease was visiting Burla. Each morning, we arrived at the laboratory to find families who had traveled during the night and saw 18 patients on the 1st day, 48 on the next, and a total of 445 patients and family members were investigated by March 4, when we ran out of the reagents for the blood cell analyzer. A further 80 subjects were screened by the electrophoresis alone.

## Findings in Odisha Patients

The study described 131 patients with homozygous SS disease (3) and 17 with sickle cell-beta^+^ thalassemia with the mutation IVSI-5G>C (4). Compared with Jamaica, where most data were derived from the cohort study, patients from Odisha were almost entirely of the Asian haplotype, had higher total hemoglobin and lower reticulocyte counts, lower mean cell volume and mean cell hemoglobin, and much higher fetal hemoglobin (HbF) levels, and over half of the patients had alpha thalassemia (Table 1).

**Table 1:** Features of patients with HbSS from Jamaica and western Odisha.

**Feature**	**Jamaica ***n*** = 311**	Odisha ***n*** = 130*	**Comments/references**
**Molecular features**			
Alpha thalassaemia	*n* = 276	*n* = 122	
α-/α-	9 (3.3%)	13 (10.7%)	(5)
α-/αα	91 (33.0%)	54 (44.3%)	
αα/αα	172 (62.3%)	55 (45.1%)	
Others	4 (1.4%)	–	
**Beta haplotype**			
Benin	80%	None	(6)
Asian	None	97%	
Red cell indices (mean, SD)**			
Fetal hemoglobin (%)	6.1, 4.2	16.6, 5.2	All differences significant at 1% level (3)
Total hemoglobin (g/dl)	8.04, 1.12	8.73, 1.69	
MCHC (g/dl)	0.34, 0.02	0.32, 0.03	
MCV (fl)	87.3, 8.1	83.6, 9.4	
MCH (pg)	29.7, 3.4	26.6, 3.6	
Reticulocytes (%)	10.5, 4.0	6.5, 4.7	
**Clinical features**			
Splenomegaly	55% in first year	Occurs later	Jamaican/Odisha data (7)
Dactylitis	45% by age 2 yrs	52% by age 5 yrs	Jamaican data (8)
Bone pain crisis	++	++	
ANFH	10–15%	15%	Jamaican data (unpublished)
Stroke	8% by 14 yrs	? 2 strokes	Jamaican data (9)
Leg ulcers	30%	<1%	Jamaican data (10)
Priapism	33%	None	Jamaican data (11)

* *One patient did not have full hematology. Jamaican data derived from the Cohort except for red cell indices*

** * which were derived from non-Cohort patients to allow better age matching*.

Clinically, there were many similarities between patients of Jamaica and India, but Indian patients differed in the rarity of chronic leg ulceration, the absence of priapism and the later appearance of splenomegaly that persisted for the longer, pitted red blood cells counts being consistent with persisting splenic function. Since the age-specificity of pneumococcal infection declines sharply after 3 years, the persistence of splenic function beyond this age could contribute to the apparent lack of pneumococcal septicemia in Indian pediatric experience. Alpha thalassemia and HbF level both inhibit sickling and their increased frequency in Odisha was consistent with a lower hemolytic rate. The impression of a milder disease in Odisha was also supported by the incidental detection of 15 patients by family study including five parents.

Dactylitis, in which avascular necrosis of the bone marrow affects small bones of the hands and feet, has clear epidemiology in Jamaican patients, commencing as early as 3 months of age, frequently recurring, usually resolving without permanent sequelae, and becoming uncommon after the age of 5 years. Recollection is likely to be unreliable but confining inquiry to the mothers of children aged 2–9 years found a prevalence similar to that among Jamaican patients. Furthermore, three Indian subjects had permanently shortened metacarpals consistent with premature fusion that occurs after an infection of the avascular bone marrow. The prevalence of avascular necrosis of the femoral head was similar between the groups although the numbers were small and influenced by symptomatic presentation. Precipitating factors for the bone pain in Odisha were similar to those in Jamaica, with almost all the patients reporting an association with skin cooling and improvement with advancing age after 30 years. It was of interest that the more frequent alpha thalassemia and high HbF levels that might be expected to inhibit intravascular sickling, did not appear to ameliorate bone pain crises, casting doubt on the relevance of these factors to this complication, but bone pain is a complex phenomenon influenced by the social, environmental, and psychological factors.

## Comparison of Odisha With Other Indian Studies

Compared to that observed in Odisha, the disease in central India has been claimed to be more severe (12, 13), but it is currently unclear whether such differences are intrinsic to the disease or different ascertainment biases in the studied populations. Geographic comparisons are fraught with difficulties in interpretation and comparing data between Nagpur and Odisha was confounded by the different age structures and ascertainment biases which, in Nagpur involved high-default rates and in Odisha included referral from existing clinics and also the chance event of hearing and responding to the radio announcements of the study. Marked elevations in HbF have been universal in Indian subjects, compared with that in Jamaica, and this may be responsible for the later appearance of splenomegaly and persistence of splenic function. The prevalence of the alpha thalassemia has varied between 16 and 55% in the Indian patients from different locations and this variation may allow assessment of the relevance of alpha thalassemia to the clinical features. Detecting populations at birth is the best method of avoiding bias, but only if the high-default rates reported from two Indian studies (13, 14) can be avoided.

## Comparison of Disease in Nagpur With Jamaican Data

Comparison of birth cohorts from Nagpur and Jamaica found that patients from Nagpur had higher HbF levels, less alpha thalassemia, few hematological differences, and later development of the splenomegaly (15).

## Return to Burla

**Figure 1:**
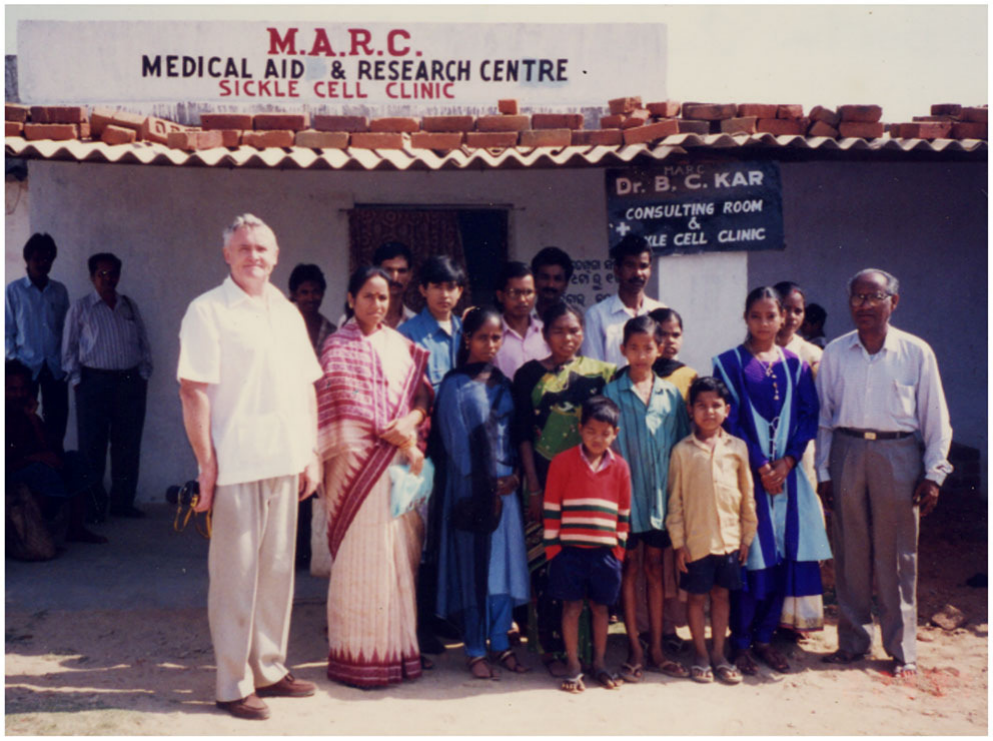
Professor Bimal Kar (right) with the patients at his post-retirement clinic in Burla Market, Odisha, India.

It was 11 years before Graham and Beryl could visit Burla again but Professor Kar continued his research interests with support from the ICMR. By 1997, Professor Kar had retired from the Burla Medical College and continued his active interest in sickle cell disease from his office in Burla market entitled MARC (Medical Aid and Research Centre) Sickle Cell Clinic (Figure 1).

In his typical dynamic and enthusiastic fashion, he had trained a local lady to take blood samples and his driver to prepare sickle tests. He passed away on September 7, 2009 having made a monumental contribution toward the clinical understanding of SS disease in India.

## Author Contributions

GS performed the histories and clinical examinations. AK did the DNA analysis. BS oversaw the hematological investigations. All authors contributed to the article and approved the submitted version.

## Conflict of Interest

The authors declare that the research was conducted in the absence of any commercial or financial relationships that could be construed as a potential conflict of interest.

## Publisher's Note

All claims expressed in this article are solely those of the authors and do not necessarily represent those of their affiliated organizations, or those of the publisher, the editors and the reviewers. Any product that may be evaluated in this article, or claim that may be made by its manufacturer, is not guaranteed or endorsed by the publisher.
